# Fraser Syndrome From Prenatal Diagnosis to Multidisciplinary Management and Long-Term Care: A Narrative Review

**DOI:** 10.7759/cureus.116461

**Published:** 2026-09-17

**Authors:** Shanza Rana

**Affiliations:** 1 Obstetrics and Gynaecology, Mid and South Essex NHS Foundation Trust, Basildon, GBR

**Keywords:** airway management, cryptophthalmos, fras1, fraser syndrome, frem2, genetic counselling, grip1, multidisciplinary care, prenatal diagnosis, syndactyly

## Abstract

Fraser syndrome (FS) is a rare autosomal recessive congenital malformation disorder characterised by cryptophthalmos, syndactyly, and variable respiratory, renal, genitourinary, craniofacial, and other systemic abnormalities. Considerable phenotypic heterogeneity creates challenges in prenatal recognition, prognostic counselling, neonatal management, and long-term care. Prenatal diagnosis may be particularly difficult because characteristic external abnormalities are not always detectable on ultrasonography, while oligohydramnios secondary to renal abnormalities may further limit fetal visualisation. The coexistence of renal agenesis and congenital high airway obstruction sequence may provide an important diagnostic clue. Following birth, airway abnormalities represent a major management challenge because laryngeal and subglottic abnormalities can result in difficult or impossible tracheal intubation. Ophthalmological, maxillofacial, dental, genetic, and other specialist input may also be required according to the individual phenotype. Although severe forms of FS are associated with poor fetal and neonatal outcomes, survival into adolescence and adulthood has been reported, shifting management priorities towards rehabilitation, function, and quality of life. This narrative review examines the current literature on FS, with particular emphasis on prenatal diagnosis, genetic assessment, airway and perioperative management, ophthalmological care, multidisciplinary rehabilitation, and long-term outcomes.

## Introduction and background

Fraser syndrome (FS), also known as cryptophthalmos-syndactyly syndrome, is a rare autosomal recessive congenital malformation disorder affecting multiple organ systems. Cryptophthalmos and cutaneous syndactyly are among its most recognisable manifestations, although abnormalities of the respiratory, renal, genitourinary, craniofacial, anorectal, and auditory systems may also occur. This broad phenotypic spectrum has been demonstrated in several large clinical reviews [1,2].

Slavotinek and Tifft reviewed 117 published cases and found cryptophthalmos in 103/117 (88%), syndactyly in 72/117 (61.5%), ear abnormalities in 69/117 (59%), renal agenesis in 53/117 (45.3%), and ambiguous genitalia in 20/117 (17.1%) [1]. A subsequent clinical study of 59 affected individuals by van Haelst et al. confirmed marked phenotypic heterogeneity and identified urinary and airway abnormalities as sufficiently frequent and clinically relevant to be incorporated among the major diagnostic criteria [2]. These findings illustrate why FS cannot be considered solely an ocular or limb malformation syndrome. Under the revised diagnostic criteria proposed by van Haelst et al., the major criteria comprise syndactyly, the cryptophthalmos spectrum, ambiguous genitalia, urinary tract abnormalities, laryngeal or tracheal anomalies, and a positive family history. Minor criteria include anorectal defects, dysplastic ears, skull ossification defects, umbilical abnormalities, and nasal anomalies. Diagnosis can be established by the presence of three major criteria, two major and two minor criteria, or one major and three minor criteria [2].

FS follows an autosomal recessive inheritance pattern. Slavotinek and Tifft reported consanguinity in 29/119 cases (24.8%) [1]. In autosomal recessive disorders, when both parents are carriers, the recurrence risk is 25% for each pregnancy, making genetic counselling particularly important for affected families.

Population-level data further demonstrate the rarity and severity of FS. In a EUROCAT population-based study of 12,886,464 births, Barisic et al. identified 26 cases, corresponding to a minimum estimated prevalence of 0.20 per 100,000 births [3]. Of these, 10/26 (38%) were liveborn, 14/26 (54%) pregnancies were terminated following prenatal detection of a serious anomaly, and 2/26 (8%) were stillborn [3]. Consequently, prevalence estimates based predominantly on live births may underestimate the true occurrence of FS [3].

FS is genetically heterogeneous. Pathogenic variants in *FRAS1*, *FREM2*, and *GRIP1* have been implicated in the disorder. *FRAS1* and *FREM2* encode components involved in embryonic epithelial-mesenchymal interactions, while GRIP1 has an important role in the localisation and function of this protein complex. Vogel et al. demonstrated pathogenic *GRIP1* variants segregating with classic FS in affected families, extending the molecular spectrum beyond *FRAS1* and *FREM2* [4].

The rarity of the syndrome means that evidence regarding management is dominated by retrospective studies, case series, case reports, and narrative reviews rather than controlled trials. The recent review by Pham et al. similarly reported consanguinity in 41% of included cases [5], further highlighting its relevance in this autosomal recessive disorder.

The multisystem nature of FS requires management to extend beyond diagnosis of cryptophthalmos and syndactyly. Clinical priorities may change substantially from fetal life to infancy and then into adolescence or adulthood. This narrative review therefore aims to examine FS across this clinical pathway, focusing on prenatal recognition, genetic assessment, neonatal and airway management, ophthalmological and supportive treatment, and long-term multidisciplinary care.

While the recent review by Pham et al. primarily synthesised the clinical spectrum and prenatal detection of FS [5], the present review addresses how management priorities evolve across the patient’s life course. In particular, it integrates perioperative and difficult-airway evidence with ophthalmological and dental rehabilitation literature and reports of long-term survivors, providing a multidisciplinary framework extending from antenatal recognition to adult care.

## Review

Literature search

A literature search was conducted using PubMed from database inception to September 2026. The principal search used the terms “Fraser syndrome” OR “cryptophthalmos-syndactyly syndrome” and retrieved 257 records. Additional topic-specific searches combined FS with terms relating to prenatal diagnosis, genetics, airway and perioperative management, renal abnormalities, ophthalmology, dental and craniofacial manifestations, rehabilitation, and long-term outcomes. Publications were selected based on their clinical relevance to the diagnosis, phenotype, genetics, prenatal assessment, management, and long-term outcomes of FS. Reviews, observational studies, case series, and clinically informative case reports were considered because of the rarity of the disorder. Articles without clinically relevant information on FS were excluded. No formal publication date restriction was applied. As this was a narrative rather than systematic review, formal meta-analysis and risk-of-bias assessment were not undertaken.

Prenatal diagnosis and genetic assessment

Prenatal recognition of FS remains challenging because some of its most characteristic postnatal manifestations are difficult to identify antenatally. Tessier et al. evaluated 38 cases and reported renal abnormalities in 37 of 38 cases, cryptophthalmos in 36 of 38, and airway abnormalities in 30 of 37 evaluable cases [6]. Among 26 pregnancies with available ultrasound information, oligohydramnios was detected in 22, renal abnormalities in 20, and evidence of high airway obstruction in 11. In contrast, syndactyly and ophthalmological abnormalities were much less frequently recognised prenatally [6].

Oligohydramnios is particularly important because it may be both a consequence of severe renal abnormalities and a barrier to accurate ultrasonographic assessment. Tessier et al. concluded that the association of oligohydramnios, renal agenesis, and congenital high airway obstruction sequence (CHAOS) should prompt consideration of FS [6].

More recent genetically confirmed cases support this association. Ikeda et al. described a fetus in whom ultrasonography demonstrated CHAOS and renal agenesis; subsequent examination identified syndactyly and cryptophthalmos, and molecular analysis revealed compound heterozygous *FREM2* variants [7]. The report also emphasised the relevance of an autosomal recessive inheritance pattern, with a 25% recurrence risk for siblings when both parents carry pathogenic variants [7].

Madan et al. similarly described prenatal suspicion of FS following identification of dysplastic kidneys, severe oligohydramnios, and features of CHAOS. Postmortem evaluation and detection of a homozygous *FRAS1* variant confirmed the diagnosis. Importantly, the authors attributed successful diagnostic evaluation and counselling to collaboration between obstetrics, fetal medicine, medical genetics, and fetal pathology [8].

These reports illustrate that prenatal management should not focus solely on obtaining a diagnostic label. Suspicion of FS has implications for genetic counselling, evaluation of recurrence risk, assessment of the severity of renal and airway abnormalities, and preparation for potential neonatal complications. Bouaoud et al. highlighted renal agenesis and laryngeal atresia as important antenatal findings [9].

Genetic testing can provide confirmation when the phenotype is uncertain and may also be valuable for counselling regarding future pregnancies. However, marked genetic and phenotypic heterogeneity means that prognosis cannot necessarily be inferred from the molecular result alone. Clinical assessment of the fetal phenotype therefore remains central to counselling and management.

Neonatal, airway and multidisciplinary management

Airway involvement is among the most clinically important aspects of FS. Reported abnormalities include laryngeal atresia, laryngeal hypoplasia, laryngeal stenosis, subglottic stenosis, and glottic webs. These abnormalities may produce respiratory compromise after birth and can make conventional tracheal intubation difficult or impossible [10-12].

The strongest perioperative evidence comes from Mathers et al., who retrospectively reviewed 125 general anaesthetics performed in 10 children with FS over a 30-year period [10]. Ten anaesthetic complications were identified, all of which were airway-related. Complications included airway obstruction, accidental extubation, post-tracheostomy bleeding, difficult intubation, and one impossible intubation requiring emergency tracheostomy. Difficult or impossible tracheal intubation occurred in 2/10 (20%) of the children, primarily because of glottic stenosis [10].

This larger perioperative series strengthens observations previously reported in individual cases. Bathla et al. described a child with known subglottic stenosis who developed worsening respiratory distress. Attempts at tracheal intubation were unsuccessful, ENT assistance was obtained, and tracheostomy was subsequently performed [11]. The authors emphasised careful pre-anaesthetic airway assessment, availability of difficult airway equipment, maintenance of appropriate airway planning, and early involvement of ENT specialists [11].

Similarly, Crowe et al. reported an infant with severe airway stenosis secondary to a congenital glottic web in whom a surgical airway was required [12]. Together, these reports demonstrate that the absence of obvious external airway abnormalities does not exclude significant laryngeal pathology. A previous history of stridor, airway obstruction, difficult intubation, tracheostomy, or documented laryngeal abnormality should therefore be carefully considered before subsequent procedures.

The available evidence does not support a single universal airway strategy because anatomy varies between affected patients [10-12]. Instead, airway planning should be tailored to the patient’s anatomy and clinical findings, with anaesthetic and ENT involvement when significant airway disease is known or suspected [10-12].

Renal abnormalities represent another major component of the FS phenotype. Renal agenesis is frequent in both large reviews and prenatal series [1-3,6]. Severity ranges from unilateral renal agenesis compatible with prolonged survival to bilateral or complex renal abnormalities associated with severe fetal disease. Renal findings therefore contribute to both prenatal suspicion and postnatal assessment.

The need for multidisciplinary care is supported by the broad range of organ systems involved. Bouaoud et al. recommended management within expert centres using collaboration between anaesthetists, ENT specialists, maxillofacial surgeons, and geneticists [9]. In practice, the exact composition of the team should reflect the individual phenotype rather than be identical for every patient.

Ophthalmological and supportive care

Cryptophthalmos remains one of the defining clinical manifestations of FS but varies considerably in severity and anatomy. Das et al. retrospectively studied 15 patients presenting to a tertiary ophthalmological centre. Bilateral involvement was more frequent than unilateral disease, and abortive cryptophthalmos was the most common subtype. Complete cryptophthalmos was associated with cystic globes, whereas abortive disease frequently involved symblepharon [13].

The clinical significance of this variability is that ophthalmological assessment must establish precise ocular and periocular anatomy rather than assuming a uniform lesion. In the Das series, some patients with abortive cryptophthalmos underwent coloboma repair for cosmetic reasons, while attempts at eyelid and ocular surface reconstruction were considered in selected complete cases where anatomical findings suggested the possibility of a formed globe [13]. This demonstrates that ophthalmological management should therefore be guided by the anatomical findings and visual potential [13].

FS can also produce substantial craniofacial and orodental morbidity. Kunz et al. evaluated dental radiographs from 10 unrelated patients and identified dental abnormalities in all of them, including hypodontia, crowding, retained teeth, and a characteristic pattern of shortened permanent tooth roots. The authors recommended integrating dentists and orthodontists into the multidisciplinary care of affected patients because these abnormalities can have a significant impact on oral health [14].

Long-term dental management may require active rehabilitation rather than surveillance alone. Amornvit et al. described a 21-year-old patient with craniofacial abnormalities and missing teeth who underwent prosthetic rehabilitation with computer-aided design/computer-aided manufacturing-fabricated overlay dentures. Follow-up demonstrated improvement in both function and aesthetics, and the authors highlighted the absence of standard oral-health guidelines for FS [15].

Hassona et al. also described oral and dental findings in a young adult with FS and noted that associated renal, cardiac, developmental, and other systemic abnormalities may affect dental treatment planning [16]. Collectively, these reports support integration of dental and maxillofacial care into the longer-term multidisciplinary pathway where relevant [14-16].

Surgical procedures for ocular, craniofacial, dental, or other manifestations must also be considered in the context of the airway abnormalities described above. The need for reconstructive or rehabilitative procedures can therefore create an interaction between specialities: an apparently elective ophthalmological or maxillofacial intervention may still require careful airway planning because of underlying laryngeal disease [10-12].

Long-term care and outcomes

The clinical course of FS is highly variable. Severe renal and respiratory manifestations can be associated with fetal or early postnatal death [3,5,6], yet survival into adolescence and adulthood is well documented [17,18]. This variability makes long-term prognosis difficult to generalise.

Koprulu et al. reported a 15.5-year-old boy with FS type 3 associated with a homozygous truncating *GRIP1* variant. He had cryptophthalmos, syndactyly, craniofacial abnormalities, and agenesis of the right kidney but no clinically significant respiratory, genital, anorectal, or umbilical abnormalities [17]. The authors considered this a non-lethal phenotype and demonstrated that even truncating *GRIP1* variants can be compatible with adolescent survival.

Rare cases demonstrate that survival can extend substantially further. Impallomeni et al. reported FS in a 96-year-old woman, illustrating the extreme end of the survival spectrum [18]. Such reports should not be interpreted as representing typical life expectancy, but they reinforce the fact that FS is not uniformly lethal and that long-term adult care may be relevant for selected patients.

The priorities of care consequently change over time. In fetal and neonatal life, diagnostic clarification, respiratory compromise, and severe renal abnormalities may dominate decision-making. In surviving children and adults, the focus may increasingly include vision, communication, oral and dental health, reconstructive needs, functional independence, psychosocial wellbeing, and quality of life.

Evidence specifically addressing quality of life in FS remains sparse. The available literature instead provides indirect evidence through individual rehabilitation reports, adult cases, and observational series. Amornvit et al. demonstrated measurable functional and aesthetic benefit from dental rehabilitation [15], while Kunz et al. identified previously under-recognised dental abnormalities with potential long-term consequences [14]. These findings support continued follow-up rather than limiting care to correction of life-threatening abnormalities during infancy.

Transition from paediatric to adult services is also likely to be relevant for long-term survivors [17,18], although formal transition pathways have not been studied. The diversity of the phenotype makes a flexible, patient-specific approach more appropriate than a rigid universal follow-up protocol.

Diagnostic criteria and historical framework

The original diagnostic framework proposed by Thomas et al. included four major criteria (cryptophthalmos, syndactyly, genital abnormalities, and an affected sibling) and eight minor criteria involving the nose, ears, larynx, oral clefts, umbilicus, kidneys, skeleton, and neurodevelopment [19]. Thomas et al. proposed a diagnosis where either two major and one minor criterion or one major and four minor criteria were present [19]. Van Haelst et al. subsequently revised these criteria, recognising urinary tract and laryngeal or tracheal abnormalities as major features [2].

Prenatal-to-perinatal airway planning

In severe cases with prenatally recognised CHAOS, delivery planning may require highly specialised airway intervention. Kohl et al. reported a fetus with FS and laryngeal-atresia-related CHAOS who underwent fetoscopic tracheal decompression and was subsequently delivered by ex-utero intrapartum treatment, allowing establishment of the airway while uteroplacental circulation was maintained. Such interventions are highly specialised and should be considered only in appropriately equipped multidisciplinary fetal therapy centres [20].

Taken together, the available literature suggests that the principal management priorities in FS change across the life course. Prenatally, renal abnormalities, oligohydramnios, and airway obstruction may provide important diagnostic and prognostic information, while severe CHAOS may require specialised perinatal airway planning. Following birth, airway assessment and renal evaluation become immediate priorities, whereas ophthalmological, craniofacial, dental, and rehabilitative needs become increasingly relevant among longer-term survivors. However, because much of the evidence derives from small series and individual case reports, management should remain phenotype-directed rather than based on a single standardised pathway.

Additional genetic considerations

FS is primarily associated with pathogenic variants in *FRAS1*, *FREM2*, and *GRIP1*. *FREM1* encodes another component of the FRAS-FREM extracellular matrix complex and is associated with clinically overlapping disorders, including Manitoba-oculo-tricho-anal (MOTA) syndrome and bifid nose, renal agenesis, and anorectal malformations (BNAR) syndrome, supporting the broader biological relevance of the FRAS-FREM pathway [21].

Limitations of the evidence and future directions

The principal limitation of the FS literature is the rarity of the disorder. Much of the evidence consists of case reports, small case series, retrospective studies, and narrative reviews. Even larger clinical publications contain relatively small numbers of patients, making robust comparison between interventions difficult.

Publication bias is also likely. Severe, unusual, or successfully treated cases may be disproportionately represented, while individuals with milder phenotypes may be underreported. Conversely, fetal cases with lethal abnormalities may be recognised only following pregnancy termination, stillbirth, or postmortem examination. These factors complicate estimates of prognosis and treatment effectiveness.

Phenotypic and genetic heterogeneity create additional difficulty. Management appropriate for a patient with unilateral renal agenesis and limited airway involvement cannot necessarily be extrapolated to a fetus with bilateral renal abnormalities and severe CHAOS. Likewise, individual case reports of successful airway, ophthalmological, or dental interventions should not be interpreted as universal treatment recommendations.

Prenatal recognition remains incomplete despite several decades of published experience [5]. Future research would benefit from multicentre registries containing standardised genetic, prenatal, surgical, renal, airway, functional, and long-term outcome data. International collaboration is particularly important because individual centres are unlikely to encounter sufficient numbers of patients to generate strong comparative evidence independently.

## Conclusions

FS is a rare multisystem disorder with substantial phenotypic variability, requiring clinical priorities to evolve across the life course. Prenatal renal abnormalities, oligohydramnios, and congenital high airway obstruction may provide important diagnostic clues, while suspected severe airway disease warrants specialist perinatal planning. After birth, airway and renal assessment are key early priorities, with ophthalmological, craniofacial, dental, and rehabilitative needs becoming increasingly relevant among survivors.

The evidence base remains limited by small observational series and case reports, preventing the development of a single standardised management pathway. Current evidence therefore supports phenotype-directed multidisciplinary care, appropriate specialist referral, and continued follow-up into adolescence and adulthood.
